# Bis(6-meth­oxy-1-methyl-2,3,4,9-tetra­hydro-1*H*-β-carbolin-2-ium) tetra­chloridozincate(II) dihydrate

**DOI:** 10.1107/S1600536812011130

**Published:** 2012-03-24

**Authors:** Teik Beng Goh, Mohd Nizam Mordi, Sharif Mahsufi Mansor, Mohd Mustaqim Rosli, Hoong-Kun Fun

**Affiliations:** a Centre for Drug Research, Universiti Sains Malaysia, 11800 USM, Penang, Malaysia; b X-ray Crystallography Unit, School of Physics, Universiti Sains Malaysia, 11800 USM, Penang, Malaysia

## Abstract

The asymmetric unit of the title compound, (C_13_H_17_N_2_O)_2_[ZnCl_4_]·2H_2_O, contains two tetra­hydro­harmine cations, one tetra­chloro­zincate(II) anion and two water mol­ecules. In the cations, the two 1*H*-indole ring systems are essentially planar, with maximum deviations of 0.016 (2) and 0.018 (2) Å, and both tetra­hydro­pyridinium rings show a half-chair conformation. The Zn^II^ complex anion has a distorted tetra­hedral geometry. In the crystal, inter­molecular N—H⋯O, N—H⋯Cl, O—H⋯O, O—H⋯Cl and C—H⋯O hydrogen bonds link the components into a three-dimensional network. A π–π inter­action with a centroid–centroid distance of 3.542 (14) Å is also observed.

## Related literature

For the biological activity of metal complexes with 6-meth­oxy-1-methyl-4,9-dihydro-3*H*-pyrido[3,4-*b*]indole, see: Al-Allaf *et al.* (1990 ▶); Herraiz *et al.* (2003 ▶). For structures of β-carboline and related compounds, see: Anlong *et al.* (2007 ▶); Larghi *et al.* (2005 ▶); Reimers *et al.* (1984 ▶); Wouters (1997 ▶); Ferretti *et al.* (2004 ▶). For a related tetra­chloridozincate structure, see: Ma *et al.* (2009 ▶). For the stability of the temperature controller used in the data collection, see: Cosier & Glazer (1986 ▶).
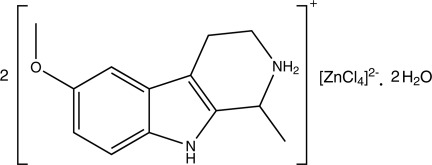


## Experimental

### 

#### Crystal data

(C_13_H_17_N_2_O)_2_[ZnCl_4_]·2H_2_O
*M* *_r_* = 677.77
Monoclinic, 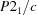

*a* = 7.3319 (1) Å
*b* = 18.5135 (3) Å
*c* = 22.0578 (3) Å
β = 91.516 (1)°
*V* = 2993.06 (8) Å^3^
*Z* = 4
Mo *K*α radiation
μ = 1.22 mm^−1^
*T* = 100 K
0.39 × 0.17 × 0.11 mm

#### Data collection

Bruker SMART APEXII CCD area-detector diffractometer
Absorption correction: multi-scan (*SADABS*; Bruker, 2009 ▶) *T*_min_ = 0.648, *T*_max_ = 0.876
33557 measured reflections
8753 independent reflections
6404 reflections with *I* > 2σ(*I*)
*R*_int_ = 0.060

#### Refinement

*R*[*F* ^2^ > 2σ(*F* ^2^)] = 0.043
*wR*(*F* ^2^) = 0.098
*S* = 1.03
8753 reflections
356 parameters
H-atom parameters constrained
Δρ_max_ = 0.70 e Å^−3^
Δρ_min_ = −0.42 e Å^−3^

### 

Data collection: *APEX2* (Bruker, 2009 ▶); cell refinement: *SAINT* (Bruker, 2009 ▶); data reduction: *SAINT*; program(s) used to solve structure: *SHELXTL* (Sheldrick, 2008 ▶); program(s) used to refine structure: *SHELXTL*; molecular graphics: *SHELXTL*; software used to prepare material for publication: *SHELXTL* and *PLATON* (Spek, 2009 ▶).

## Supplementary Material

Crystal structure: contains datablock(s) I, global. DOI: 10.1107/S1600536812011130/is5085sup1.cif

Structure factors: contains datablock(s) I. DOI: 10.1107/S1600536812011130/is5085Isup2.hkl

Additional supplementary materials:  crystallographic information; 3D view; checkCIF report

## Figures and Tables

**Table 1:** Hydrogen-bond geometry (Å, °)

*D*—H⋯*A*	*D*—H	H⋯*A*	*D*⋯*A*	*D*—H⋯*A*
N1*A*—H1*NA*⋯Cl3^i^	0.90	2.49	3.271 (2)	146
N2*A*—H2*NA*⋯Cl4^ii^	0.80	2.43	3.208 (2)	164
N2*A*—H3*NA*⋯O1*WB*^iii^	0.89	1.92	2.790 (3)	166
N1*B*—H1*NB*⋯O1*WB*	0.92	1.99	2.872 (3)	159
N2*B*—H2*NB*⋯Cl1^iii^	0.86	2.28	3.141 (2)	176
N2*B*—H3*NB*⋯Cl3^ii^	0.92	2.32	3.231 (2)	171
O1*WA*—H1*WA*⋯Cl4^iv^	0.92	2.42	3.204 (2)	143
O1*WA*—H2*WA*⋯O1*A*^v^	0.91	1.89	2.799 (3)	173
O1*WB*—H1*WB*⋯O1*WA*^iv^	0.90	1.78	2.672 (3)	175
O1*WB*—H2*WB*⋯Cl2	0.91	2.33	3.231 (2)	169
C12*B*—H12*E*⋯O1*WA*^vi^	0.98	2.58	3.487 (3)	153
C3*B*—H3*BA*⋯O1*B*^vii^	0.95	2.56	3.373 (3)	143

Symmetry codes: (i) 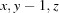; (ii) 
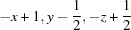
; (iii) 
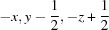
; (iv) 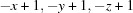; (v) 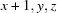; (vi) 
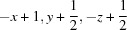
; (vii) 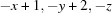.

**Fig. 1.**
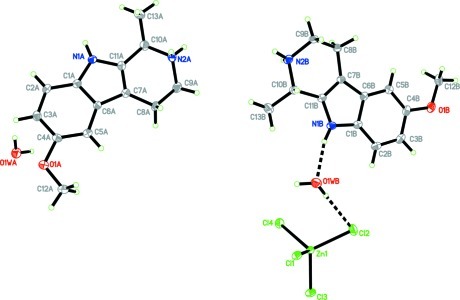
The molecular structure of the title compound, showing 50% probability displacement ellipsoids. Hydrogen atoms are shown as spheres of arbitrary radius.

**Fig. 2.**
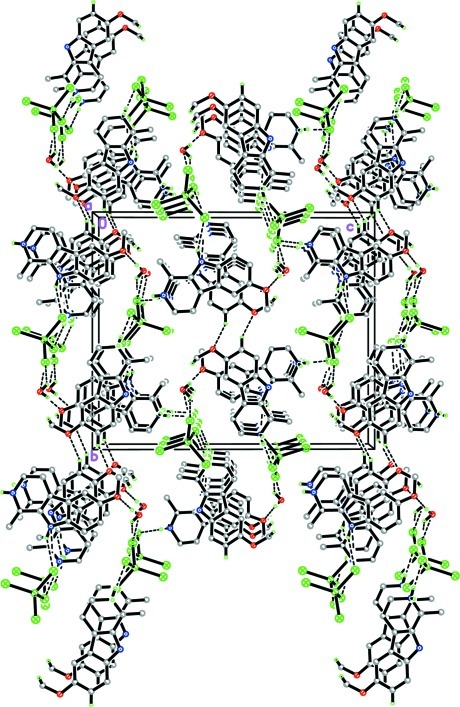
A crystal packing diagram of the title compound. Dashed lines indicate hydrogen bonds. H atoms not involved in the hydrogen bond interactions have been omitted for clarity.

## supplementary crystallographic information

### Comment

The metal complexes of the 6-methoxy-1-methyl-4,9-dihydro-3*H*-β-carboline and other carboline alkaloids was previously reported to have biological activity (Al-Allaf *et al.*, 1990). It is now well established that these classes of beta carboline alkaloids may occur under mild conditions in foods from a Pictet Spengler condensation of indoleamines such as *L*-tryptophan and short aliphatic aldehydes (Herraiz *et al.*, 2003). Our present work intend to synthesize the titled compound and prepare it in salt form to investigate its safety and antiproliferative efficacy in cancer cell line.

The asymmetric unit of I contains two tetrahydroharmine cations, one zinc tetrachloride anion and two water molecules (Fig. 1). In these tetrahydroharmine cations, the maximum deviations for the two 1H-indole planes are 0.016 (2) and 0.018 (2) Å, respectively, for molecules A and B. The tetrahydropyridinium rings in both molecules show a half-chair conformation with the puckering parameters Q = 0.488 (3) Å, θ = 48.9 (4)°, φ = 24.3 (4)° for molecule A, and Q = 0.504 (3) Å, θ = 130.7 (3)°, φ = 206.6 (4)° for molecule B. The crystal structure has extensive intermolecular N—H···C, N—H···O, O—H···Cl, O—H···O and C—H···O interactions (Table 1) that link the three components into a three-dimensional network (Fig. 2). A π–π interaction with a centroid-centroid distance of 3.542 (14) Å is observed (*Cg*1 = N1A—C11A, *Cg*2 = C1B—C6B, -*x*, *y* + 1, -*z* + 1/2).

### Experimental

2*M* solution of hydrochloric acid in methanol was prepared by transferring 1.68 ml of 37% concentrated HCl acid into a 10 ml volumetric flask and was topped up to volume using methanol. The ZnCl_2_ (1 mmol, 136 mg) was weighed and 0.50 ml of 2*M* hydrochloric acid in methanol was added. Sonication was performed to aid dissolution. 6-methoxy-1-methyl-4,9-dihydro-3*H*-β-carboline (2.5 mmol, 540 mg) was weighed and 0.50 ml HPLC grade methanol was added. The solution was heated on water bath to facilitate dissolution. 0.50 ml of the ZnCl_2_ solution in 2*M* hydrochloric acid was then added to the 6-methoxy-1-methyl-4,9-dihydro-3*H*-β-carboline solution in methanol dropwise in a glass bottle. The side of the glass bottle was scratched with a small spatula and the bottle was kept in fridge at 4 °C for 5–7 days before yielding the colourless crystals of Bis(6-methoxy-1-methyl-4,9-dihydro-3*H*-β-carbolinium) tetrachloridozincatedihydrate which were filtered off, washed twice with acetone and air-dried. Crystals of the title compound, suitable to X-ray diffraction analysis, were selected directly from the samples as prepared.

### Refinement

O and N bound H atoms are located from a difference Fourier map and refined using a riding model. The remaining H atoms were positioned geometrically and refined using a riding model with C—H = 0.95–1.00 Å and *U*_iso_(H) = 1.2 or 1.5*U*_eq_(C-methyl). A rotating group model was applied to the methyl groups.

### Crystal data

**Table:**

(C_13_H_17_N_2_O)_2_[ZnCl_4_]·2H_2_O	*F*(000) = 1408
*M**_r_* = 677.77	*D*_x_ = 1.504 Mg m^−^^3^
Monoclinic, *P*2_1_/*c*	Mo *K*α radiation, λ = 0.71073 Å
Hall symbol: -P 2ybc	Cell parameters from 6348 reflections
*a* = 7.3319 (1) Å	θ = 2.4–30.0°
*b* = 18.5135 (3) Å	µ = 1.22 mm^−^^1^
*c* = 22.0578 (3) Å	*T* = 100 K
β = 91.516 (1)°	Block, colourless
*V* = 2993.06 (8) Å^3^	0.39 × 0.17 × 0.11 mm
*Z* = 4	

### Data collection

**Table:**

Bruker SMART APEXII CCD area-detector diffractometer	8753 independent reflections
Radiation source: fine-focus sealed tube	6404 reflections with *I* > 2σ(*I*)
Graphite monochromator	*R*_int_ = 0.060
φ and ω scans	θ_max_ = 30.1°, θ_min_ = 2.2°
Absorption correction: multi-scan (*SADABS*; Bruker, 2009)	*h* = −10→10
*T*_min_ = 0.648, *T*_max_ = 0.876	*k* = −25→26
33557 measured reflections	*l* = −29→31

### Refinement

**Table:**

Refinement on *F*^2^	Primary atom site location: structure-invariant direct methods
Least-squares matrix: full	Secondary atom site location: difference Fourier map
*R*[*F*^2^ > 2σ(*F*^2^)] = 0.043	Hydrogen site location: inferred from neighbouring sites
*wR*(*F*^2^) = 0.098	H-atom parameters constrained
*S* = 1.03	*w* = 1/[σ^2^(*F*_o_^2^) + (0.034*P*)^2^ + 2.4492*P*] where *P* = (*F*_o_^2^ + 2*F*_c_^2^)/3
8753 reflections	(Δ/σ)_max_ = 0.001
356 parameters	Δρ_max_ = 0.70 e Å^−^^3^
0 restraints	Δρ_min_ = −0.42 e Å^−^^3^

### Special details

**Table:**

Experimental. The crystal was placed in the cold stream of an Oxford Cryosystems Cobra open-flow nitrogen cryostat (Cosier & Glazer, 1986) operating at 100.0 (1) K.
Geometry. All e.s.d.'s (except the e.s.d. in the dihedral angle between two l.s. planes) are estimated using the full covariance matrix. The cell e.s.d.'s are taken into account individually in the estimation of e.s.d.'s in distances, angles and torsion angles; correlations between e.s.d.'s in cell parameters are only used when they are defined by crystal symmetry. An approximate (isotropic) treatment of cell e.s.d.'s is used for estimating e.s.d.'s involving l.s. planes.
Refinement. Refinement of *F*^2^ against ALL reflections. The weighted *R*-factor *wR* and goodness of fit *S* are based on *F*^2^, conventional *R*-factors *R* are based on *F*, with *F* set to zero for negative *F*^2^. The threshold expression of *F*^2^ > σ(*F*^2^) is used only for calculating *R*-factors(gt) *etc*. and is not relevant to the choice of reflections for refinement. *R*-factors based on *F*^2^ are statistically about twice as large as those based on *F*, and *R*- factors based on ALL data will be even larger.

### Fractional atomic coordinates and isotropic or equivalent isotropic displacement parameters (Å^2^)

**Table:**

	*x*	*y*	*z*	*U*_iso_*/*U*_eq_	
Zn1	0.26573 (4)	0.962442 (15)	0.338408 (12)	0.01473 (7)	
Cl1	−0.01952 (8)	0.92451 (3)	0.36215 (3)	0.01755 (12)	
Cl2	0.24649 (10)	0.99951 (4)	0.24105 (3)	0.02531 (15)	
Cl3	0.34993 (9)	1.05252 (3)	0.40340 (3)	0.02075 (13)	
Cl4	0.47209 (9)	0.87115 (3)	0.34904 (3)	0.02156 (13)	
O1A	0.0570 (3)	0.33708 (9)	0.60925 (7)	0.0208 (4)	
N1A	0.2953 (3)	0.22751 (11)	0.39336 (9)	0.0168 (4)	
H1NA	0.3328	0.1841	0.3806	0.020*	
N2A	0.2749 (3)	0.35932 (11)	0.26700 (9)	0.0206 (5)	
H2NA	0.3187	0.3633	0.2343	0.025*	
H3NA	0.1567	0.3510	0.2614	0.025*	
C1A	0.2379 (3)	0.24608 (13)	0.45051 (11)	0.0155 (5)	
C2A	0.2228 (3)	0.20436 (13)	0.50269 (11)	0.0176 (5)	
H2AA	0.2534	0.1545	0.5027	0.021*	
C3A	0.1624 (3)	0.23764 (13)	0.55412 (11)	0.0183 (5)	
H3AA	0.1514	0.2104	0.5904	0.022*	
C4A	0.1165 (3)	0.31170 (13)	0.55381 (11)	0.0161 (5)	
C5A	0.1324 (3)	0.35399 (13)	0.50265 (11)	0.0159 (5)	
H5AA	0.1023	0.4039	0.5031	0.019*	
C6A	0.1949 (3)	0.32041 (12)	0.44983 (10)	0.0144 (5)	
C7A	0.2315 (3)	0.34639 (13)	0.39013 (11)	0.0158 (5)	
C8A	0.2124 (4)	0.42097 (13)	0.36420 (11)	0.0185 (5)	
H8AA	0.2649	0.4567	0.3932	0.022*	
H8AB	0.0817	0.4325	0.3573	0.022*	
C9A	0.3114 (4)	0.42501 (14)	0.30467 (12)	0.0238 (6)	
H9AA	0.2706	0.4685	0.2819	0.029*	
H9AB	0.4442	0.4295	0.3131	0.029*	
C10A	0.3524 (4)	0.29054 (14)	0.29338 (11)	0.0207 (5)	
H10A	0.4886	0.2933	0.2932	0.025*	
C11A	0.2923 (3)	0.28910 (13)	0.35792 (11)	0.0171 (5)	
C12A	0.0208 (4)	0.41291 (14)	0.61312 (12)	0.0230 (5)	
H12A	−0.0174	0.4248	0.6542	0.034*	
H12B	−0.0766	0.4259	0.5838	0.034*	
H12C	0.1316	0.4400	0.6040	0.034*	
C13A	0.2905 (4)	0.22674 (15)	0.25513 (12)	0.0269 (6)	
H13A	0.3437	0.1823	0.2720	0.040*	
H13B	0.3306	0.2333	0.2135	0.040*	
H13C	0.1571	0.2234	0.2552	0.040*	
O1B	0.4468 (3)	0.92386 (9)	−0.07206 (8)	0.0229 (4)	
N1B	0.2155 (3)	0.76370 (11)	0.12185 (9)	0.0168 (4)	
H1NB	0.1594	0.7771	0.1571	0.020*	
N2B	0.2106 (3)	0.56588 (11)	0.09296 (9)	0.0180 (4)	
H2NB	0.1558	0.5268	0.1035	0.022*	
H3NB	0.3344	0.5618	0.0984	0.022*	
C1B	0.2720 (3)	0.81144 (13)	0.07788 (11)	0.0159 (5)	
C2B	0.2923 (3)	0.88670 (13)	0.07965 (11)	0.0191 (5)	
H2BA	0.2675	0.9133	0.1153	0.023*	
C3B	0.3491 (3)	0.92074 (13)	0.02824 (12)	0.0205 (5)	
H3BA	0.3615	0.9718	0.0282	0.025*	
C4B	0.3893 (3)	0.88144 (13)	−0.02451 (11)	0.0184 (5)	
C5B	0.3702 (3)	0.80745 (13)	−0.02703 (11)	0.0164 (5)	
H5BA	0.3975	0.7813	−0.0627	0.020*	
C6B	0.3089 (3)	0.77191 (12)	0.02494 (11)	0.0147 (5)	
C7B	0.2684 (3)	0.69762 (12)	0.03832 (10)	0.0142 (4)	
C8B	0.2740 (4)	0.63242 (12)	−0.00178 (11)	0.0169 (5)	
H8BA	0.4020	0.6169	−0.0066	0.020*	
H8BB	0.2218	0.6443	−0.0424	0.020*	
C9B	0.1652 (4)	0.57181 (13)	0.02624 (11)	0.0183 (5)	
H9BA	0.0331	0.5814	0.0202	0.022*	
H9BB	0.1937	0.5256	0.0060	0.022*	
C10B	0.1468 (4)	0.62980 (13)	0.12931 (11)	0.0185 (5)	
H10B	0.0104	0.6301	0.1288	0.022*	
C11B	0.2120 (3)	0.69530 (12)	0.09674 (11)	0.0151 (5)	
C12B	0.4831 (4)	0.88787 (15)	−0.12753 (11)	0.0237 (6)	
H12D	0.5254	0.9230	−0.1572	0.036*	
H12E	0.3713	0.8647	−0.1431	0.036*	
H12F	0.5775	0.8512	−0.1204	0.036*	
C13B	0.2165 (4)	0.62126 (15)	0.19426 (11)	0.0264 (6)	
H13D	0.1824	0.6638	0.2179	0.040*	
H13E	0.3497	0.6165	0.1949	0.040*	
H13F	0.1626	0.5780	0.2120	0.040*	
O1WA	0.8196 (3)	0.25435 (10)	0.67829 (9)	0.0288 (4)	
H1WA	0.7555	0.2236	0.6529	0.043*	
H2WA	0.9016	0.2780	0.6552	0.043*	
O1WB	0.1004 (3)	0.83525 (10)	0.22985 (8)	0.0238 (4)	
H1WB	0.1204	0.8048	0.2610	0.036*	
H2WB	0.1576	0.8790	0.2333	0.036*	

### Atomic displacement parameters (Å^2^)

**Table:**

	*U*^11^	*U*^22^	*U*^33^	*U*^12^	*U*^13^	*U*^23^
Zn1	0.01490 (14)	0.01365 (13)	0.01579 (13)	−0.00034 (11)	0.00312 (10)	−0.00027 (11)
Cl1	0.0148 (3)	0.0147 (3)	0.0234 (3)	−0.0003 (2)	0.0051 (2)	−0.0018 (2)
Cl2	0.0292 (4)	0.0285 (3)	0.0183 (3)	0.0008 (3)	0.0031 (3)	0.0068 (2)
Cl3	0.0213 (3)	0.0158 (3)	0.0253 (3)	−0.0025 (2)	0.0016 (2)	−0.0045 (2)
Cl4	0.0188 (3)	0.0236 (3)	0.0227 (3)	0.0068 (2)	0.0071 (2)	0.0033 (2)
O1A	0.0264 (10)	0.0188 (9)	0.0174 (8)	−0.0001 (7)	0.0028 (7)	−0.0022 (7)
N1A	0.0194 (11)	0.0140 (9)	0.0170 (10)	0.0017 (8)	0.0025 (8)	−0.0022 (8)
N2A	0.0198 (11)	0.0235 (11)	0.0187 (10)	−0.0003 (9)	0.0060 (9)	0.0052 (9)
C1A	0.0131 (11)	0.0148 (11)	0.0188 (11)	−0.0012 (9)	0.0022 (9)	−0.0009 (9)
C2A	0.0180 (12)	0.0133 (11)	0.0216 (12)	−0.0014 (9)	0.0001 (10)	0.0007 (9)
C3A	0.0169 (12)	0.0197 (12)	0.0181 (12)	−0.0028 (10)	−0.0003 (10)	0.0027 (9)
C4A	0.0126 (11)	0.0198 (12)	0.0159 (11)	−0.0026 (9)	−0.0003 (9)	−0.0039 (9)
C5A	0.0131 (11)	0.0149 (11)	0.0197 (11)	0.0003 (9)	−0.0002 (9)	−0.0014 (9)
C6A	0.0131 (11)	0.0138 (11)	0.0162 (11)	−0.0006 (9)	−0.0002 (9)	0.0003 (9)
C7A	0.0133 (11)	0.0157 (11)	0.0184 (11)	0.0000 (9)	0.0014 (9)	0.0007 (9)
C8A	0.0209 (13)	0.0146 (11)	0.0201 (12)	0.0002 (10)	0.0027 (10)	0.0033 (9)
C9A	0.0276 (15)	0.0175 (12)	0.0265 (13)	−0.0042 (11)	0.0062 (11)	0.0026 (10)
C10A	0.0208 (13)	0.0217 (12)	0.0199 (12)	0.0037 (10)	0.0057 (10)	0.0012 (10)
C11A	0.0167 (12)	0.0175 (11)	0.0169 (11)	−0.0006 (9)	0.0003 (9)	0.0019 (9)
C12A	0.0260 (14)	0.0220 (13)	0.0210 (12)	0.0003 (11)	0.0016 (11)	−0.0061 (10)
C13A	0.0337 (16)	0.0270 (14)	0.0200 (13)	0.0043 (12)	0.0016 (11)	−0.0026 (11)
O1B	0.0268 (10)	0.0170 (9)	0.0251 (9)	−0.0034 (8)	0.0033 (8)	0.0067 (7)
N1B	0.0173 (11)	0.0159 (10)	0.0174 (10)	−0.0013 (8)	0.0049 (8)	−0.0034 (8)
N2B	0.0201 (11)	0.0141 (9)	0.0202 (10)	−0.0042 (8)	0.0044 (8)	0.0002 (8)
C1B	0.0120 (11)	0.0157 (11)	0.0199 (12)	−0.0002 (9)	−0.0003 (9)	−0.0020 (9)
C2B	0.0174 (12)	0.0161 (11)	0.0239 (13)	0.0016 (9)	−0.0003 (10)	−0.0051 (10)
C3B	0.0171 (13)	0.0143 (11)	0.0299 (13)	0.0005 (9)	−0.0020 (10)	0.0014 (10)
C4B	0.0135 (11)	0.0170 (11)	0.0246 (12)	−0.0021 (9)	−0.0004 (10)	0.0056 (10)
C5B	0.0127 (11)	0.0179 (11)	0.0186 (11)	−0.0002 (9)	0.0011 (9)	0.0005 (9)
C6B	0.0108 (11)	0.0143 (11)	0.0190 (11)	0.0012 (9)	−0.0001 (9)	0.0005 (9)
C7B	0.0133 (11)	0.0142 (11)	0.0150 (11)	−0.0012 (9)	0.0006 (9)	−0.0015 (9)
C8B	0.0200 (12)	0.0158 (11)	0.0150 (11)	−0.0025 (9)	0.0030 (9)	−0.0004 (9)
C9B	0.0225 (13)	0.0141 (11)	0.0183 (12)	−0.0032 (10)	0.0016 (10)	−0.0026 (9)
C10B	0.0199 (13)	0.0173 (11)	0.0185 (12)	−0.0032 (10)	0.0051 (10)	−0.0022 (9)
C11B	0.0130 (11)	0.0143 (11)	0.0181 (11)	−0.0009 (9)	0.0024 (9)	−0.0015 (9)
C12B	0.0234 (14)	0.0252 (13)	0.0225 (13)	−0.0031 (11)	0.0013 (11)	0.0082 (11)
C13B	0.0355 (16)	0.0261 (14)	0.0179 (12)	−0.0035 (12)	0.0032 (11)	0.0007 (10)
O1WA	0.0327 (12)	0.0249 (10)	0.0289 (10)	−0.0012 (9)	0.0048 (9)	0.0014 (8)
O1WB	0.0274 (10)	0.0238 (9)	0.0202 (9)	−0.0009 (8)	0.0034 (8)	−0.0021 (7)

### Geometric parameters (Å, º)

**Table:**

Zn1—Cl2	2.2554 (7)	O1B—C4B	1.385 (3)
Zn1—Cl3	2.2739 (7)	O1B—C12B	1.425 (3)
Zn1—Cl4	2.2763 (7)	N1B—C11B	1.382 (3)
Zn1—Cl1	2.2802 (6)	N1B—C1B	1.384 (3)
O1A—C4A	1.391 (3)	N1B—H1NB	0.9238
O1A—C12A	1.432 (3)	N2B—C9B	1.504 (3)
N1A—C11A	1.382 (3)	N2B—C10B	1.511 (3)
N1A—C1A	1.383 (3)	N2B—H2NB	0.8624
N1A—H1NA	0.8963	N2B—H3NB	0.9155
N2A—C9A	1.493 (3)	C1B—C2B	1.402 (3)
N2A—C10A	1.505 (3)	C1B—C6B	1.410 (3)
N2A—H2NA	0.8004	C2B—C3B	1.372 (3)
N2A—H3NA	0.8857	C2B—H2BA	0.9500
C1A—C2A	1.393 (3)	C3B—C4B	1.410 (4)
C1A—C6A	1.412 (3)	C3B—H3BA	0.9500
C2A—C3A	1.375 (3)	C4B—C5B	1.378 (3)
C2A—H2AA	0.9500	C5B—C6B	1.406 (3)
C3A—C4A	1.412 (3)	C5B—H5BA	0.9500
C3A—H3AA	0.9500	C6B—C7B	1.439 (3)
C4A—C5A	1.381 (3)	C7B—C11B	1.364 (3)
C5A—C6A	1.408 (3)	C7B—C8B	1.498 (3)
C5A—H5AA	0.9500	C8B—C9B	1.518 (3)
C6A—C7A	1.434 (3)	C8B—H8BA	0.9900
C7A—C11A	1.358 (3)	C8B—H8BB	0.9900
C7A—C8A	1.500 (3)	C9B—H9BA	0.9900
C8A—C9A	1.519 (3)	C9B—H9BB	0.9900
C8A—H8AA	0.9900	C10B—C11B	1.495 (3)
C8A—H8AB	0.9900	C10B—C13B	1.517 (4)
C9A—H9AA	0.9900	C10B—H10B	1.0000
C9A—H9AB	0.9900	C12B—H12D	0.9800
C10A—C11A	1.501 (3)	C12B—H12E	0.9800
C10A—C13A	1.514 (4)	C12B—H12F	0.9800
C10A—H10A	1.0000	C13B—H13D	0.9800
C12A—H12A	0.9800	C13B—H13E	0.9800
C12A—H12B	0.9800	C13B—H13F	0.9800
C12A—H12C	0.9800	O1WA—H1WA	0.9187
C13A—H13A	0.9800	O1WA—H2WA	0.9097
C13A—H13B	0.9800	O1WB—H1WB	0.8973
C13A—H13C	0.9800	O1WB—H2WB	0.9144
			
Cl2—Zn1—Cl3	112.72 (3)	H13A—C13A—H13C	109.5
Cl2—Zn1—Cl4	110.40 (3)	H13B—C13A—H13C	109.5
Cl3—Zn1—Cl4	108.12 (3)	C4B—O1B—C12B	116.91 (19)
Cl2—Zn1—Cl1	106.12 (3)	C11B—N1B—C1B	107.89 (19)
Cl3—Zn1—Cl1	108.45 (2)	C11B—N1B—H1NB	125.4
Cl4—Zn1—Cl1	111.03 (2)	C1B—N1B—H1NB	124.6
C4A—O1A—C12A	116.52 (19)	C9B—N2B—C10B	113.50 (19)
C11A—N1A—C1A	108.04 (19)	C9B—N2B—H2NB	103.4
C11A—N1A—H1NA	124.2	C10B—N2B—H2NB	111.2
C1A—N1A—H1NA	127.7	C9B—N2B—H3NB	109.1
C9A—N2A—C10A	114.4 (2)	C10B—N2B—H3NB	108.4
C9A—N2A—H2NA	110.9	H2NB—N2B—H3NB	111.2
C10A—N2A—H2NA	105.6	N1B—C1B—C2B	130.5 (2)
C9A—N2A—H3NA	112.3	N1B—C1B—C6B	108.4 (2)
C10A—N2A—H3NA	105.3	C2B—C1B—C6B	121.1 (2)
H2NA—N2A—H3NA	107.8	C3B—C2B—C1B	117.9 (2)
N1A—C1A—C2A	130.4 (2)	C3B—C2B—H2BA	121.0
N1A—C1A—C6A	107.8 (2)	C1B—C2B—H2BA	121.0
C2A—C1A—C6A	121.8 (2)	C2B—C3B—C4B	121.3 (2)
C3A—C2A—C1A	117.9 (2)	C2B—C3B—H3BA	119.3
C3A—C2A—H2AA	121.1	C4B—C3B—H3BA	119.3
C1A—C2A—H2AA	121.1	C5B—C4B—O1B	124.5 (2)
C2A—C3A—C4A	120.9 (2)	C5B—C4B—C3B	121.5 (2)
C2A—C3A—H3AA	119.6	O1B—C4B—C3B	114.0 (2)
C4A—C3A—H3AA	119.6	C4B—C5B—C6B	117.8 (2)
C5A—C4A—O1A	124.2 (2)	C4B—C5B—H5BA	121.1
C5A—C4A—C3A	122.0 (2)	C6B—C5B—H5BA	121.1
O1A—C4A—C3A	113.8 (2)	C5B—C6B—C1B	120.3 (2)
C4A—C5A—C6A	117.5 (2)	C5B—C6B—C7B	133.4 (2)
C4A—C5A—H5AA	121.2	C1B—C6B—C7B	106.3 (2)
C6A—C5A—H5AA	121.2	C11B—C7B—C6B	107.0 (2)
C5A—C6A—C1A	119.9 (2)	C11B—C7B—C8B	123.1 (2)
C5A—C6A—C7A	133.2 (2)	C6B—C7B—C8B	129.8 (2)
C1A—C6A—C7A	106.8 (2)	C7B—C8B—C9B	109.41 (19)
C11A—C7A—C6A	106.9 (2)	C7B—C8B—H8BA	109.8
C11A—C7A—C8A	123.2 (2)	C9B—C8B—H8BA	109.8
C6A—C7A—C8A	129.9 (2)	C7B—C8B—H8BB	109.8
C7A—C8A—C9A	109.5 (2)	C9B—C8B—H8BB	109.8
C7A—C8A—H8AA	109.8	H8BA—C8B—H8BB	108.2
C9A—C8A—H8AA	109.8	N2B—C9B—C8B	110.3 (2)
C7A—C8A—H8AB	109.8	N2B—C9B—H9BA	109.6
C9A—C8A—H8AB	109.8	C8B—C9B—H9BA	109.6
H8AA—C8A—H8AB	108.2	N2B—C9B—H9BB	109.6
N2A—C9A—C8A	111.0 (2)	C8B—C9B—H9BB	109.6
N2A—C9A—H9AA	109.4	H9BA—C9B—H9BB	108.1
C8A—C9A—H9AA	109.4	C11B—C10B—N2B	105.84 (18)
N2A—C9A—H9AB	109.4	C11B—C10B—C13B	115.8 (2)
C8A—C9A—H9AB	109.4	N2B—C10B—C13B	108.6 (2)
H9AA—C9A—H9AB	108.0	C11B—C10B—H10B	108.8
C11A—C10A—N2A	105.33 (19)	N2B—C10B—H10B	108.8
C11A—C10A—C13A	115.0 (2)	C13B—C10B—H10B	108.8
N2A—C10A—C13A	109.9 (2)	C7B—C11B—N1B	110.3 (2)
C11A—C10A—H10A	108.8	C7B—C11B—C10B	126.0 (2)
N2A—C10A—H10A	108.8	N1B—C11B—C10B	123.6 (2)
C13A—C10A—H10A	108.8	O1B—C12B—H12D	109.5
C7A—C11A—N1A	110.4 (2)	O1B—C12B—H12E	109.5
C7A—C11A—C10A	126.2 (2)	H12D—C12B—H12E	109.5
N1A—C11A—C10A	123.4 (2)	O1B—C12B—H12F	109.5
O1A—C12A—H12A	109.5	H12D—C12B—H12F	109.5
O1A—C12A—H12B	109.5	H12E—C12B—H12F	109.5
H12A—C12A—H12B	109.5	C10B—C13B—H13D	109.5
O1A—C12A—H12C	109.5	C10B—C13B—H13E	109.5
H12A—C12A—H12C	109.5	H13D—C13B—H13E	109.5
H12B—C12A—H12C	109.5	C10B—C13B—H13F	109.5
C10A—C13A—H13A	109.5	H13D—C13B—H13F	109.5
C10A—C13A—H13B	109.5	H13E—C13B—H13F	109.5
H13A—C13A—H13B	109.5	H1WA—O1WA—H2WA	107.0
C10A—C13A—H13C	109.5	H1WB—O1WB—H2WB	115.3
			
C11A—N1A—C1A—C2A	−177.6 (3)	C11B—N1B—C1B—C2B	177.7 (3)
C11A—N1A—C1A—C6A	1.2 (3)	C11B—N1B—C1B—C6B	−1.5 (3)
N1A—C1A—C2A—C3A	179.4 (2)	N1B—C1B—C2B—C3B	−179.1 (3)
C6A—C1A—C2A—C3A	0.7 (4)	C6B—C1B—C2B—C3B	0.0 (4)
C1A—C2A—C3A—C4A	0.3 (4)	C1B—C2B—C3B—C4B	−1.2 (4)
C12A—O1A—C4A—C5A	−3.6 (3)	C12B—O1B—C4B—C5B	1.9 (4)
C12A—O1A—C4A—C3A	175.7 (2)	C12B—O1B—C4B—C3B	−177.7 (2)
C2A—C3A—C4A—C5A	−1.0 (4)	C2B—C3B—C4B—C5B	1.2 (4)
C2A—C3A—C4A—O1A	179.7 (2)	C2B—C3B—C4B—O1B	−179.3 (2)
O1A—C4A—C5A—C6A	179.9 (2)	O1B—C4B—C5B—C6B	−179.5 (2)
C3A—C4A—C5A—C6A	0.7 (4)	C3B—C4B—C5B—C6B	0.0 (4)
C4A—C5A—C6A—C1A	0.2 (3)	C4B—C5B—C6B—C1B	−1.1 (4)
C4A—C5A—C6A—C7A	−178.5 (3)	C4B—C5B—C6B—C7B	177.8 (3)
N1A—C1A—C6A—C5A	−179.9 (2)	N1B—C1B—C6B—C5B	−179.6 (2)
C2A—C1A—C6A—C5A	−0.9 (4)	C2B—C1B—C6B—C5B	1.1 (4)
N1A—C1A—C6A—C7A	−0.9 (3)	N1B—C1B—C6B—C7B	1.2 (3)
C2A—C1A—C6A—C7A	178.1 (2)	C2B—C1B—C6B—C7B	−178.1 (2)
C5A—C6A—C7A—C11A	179.1 (3)	C5B—C6B—C7B—C11B	−179.5 (3)
C1A—C6A—C7A—C11A	0.2 (3)	C1B—C6B—C7B—C11B	−0.4 (3)
C5A—C6A—C7A—C8A	−0.5 (5)	C5B—C6B—C7B—C8B	−2.2 (5)
C1A—C6A—C7A—C8A	−179.3 (2)	C1B—C6B—C7B—C8B	176.8 (2)
C11A—C7A—C8A—C9A	−13.4 (3)	C11B—C7B—C8B—C9B	14.5 (3)
C6A—C7A—C8A—C9A	166.1 (3)	C6B—C7B—C8B—C9B	−162.4 (2)
C10A—N2A—C9A—C8A	−66.4 (3)	C10B—N2B—C9B—C8B	67.6 (3)
C7A—C8A—C9A—N2A	43.0 (3)	C7B—C8B—C9B—N2B	−45.0 (3)
C9A—N2A—C10A—C11A	49.5 (3)	C9B—N2B—C10B—C11B	−49.5 (3)
C9A—N2A—C10A—C13A	174.0 (2)	C9B—N2B—C10B—C13B	−174.4 (2)
C6A—C7A—C11A—N1A	0.5 (3)	C6B—C7B—C11B—N1B	−0.5 (3)
C8A—C7A—C11A—N1A	−179.9 (2)	C8B—C7B—C11B—N1B	−178.0 (2)
C6A—C7A—C11A—C10A	−178.3 (2)	C6B—C7B—C11B—C10B	176.8 (2)
C8A—C7A—C11A—C10A	1.2 (4)	C8B—C7B—C11B—C10B	−0.7 (4)
C1A—N1A—C11A—C7A	−1.1 (3)	C1B—N1B—C11B—C7B	1.3 (3)
C1A—N1A—C11A—C10A	177.8 (2)	C1B—N1B—C11B—C10B	−176.1 (2)
N2A—C10A—C11A—C7A	−18.0 (3)	N2B—C10B—C11B—C7B	17.2 (3)
C13A—C10A—C11A—C7A	−139.1 (3)	C13B—C10B—C11B—C7B	137.5 (3)
N2A—C10A—C11A—N1A	163.3 (2)	N2B—C10B—C11B—N1B	−165.8 (2)
C13A—C10A—C11A—N1A	42.1 (4)	C13B—C10B—C11B—N1B	−45.5 (3)

### Hydrogen-bond geometry (Å, º)

**Table:**

*D*—H···*A*	*D*—H	H···*A*	*D*···*A*	*D*—H···*A*
N1*A*—H1*NA*···Cl3^i^	0.90	2.49	3.271 (2)	146
N2*A*—H2*NA*···Cl4^ii^	0.80	2.43	3.208 (2)	164
N2*A*—H3*NA*···O1*WB*^iii^	0.89	1.92	2.790 (3)	166
N1*B*—H1*NB*···O1*WB*	0.92	1.99	2.872 (3)	159
N2*B*—H2*NB*···Cl1^iii^	0.86	2.28	3.141 (2)	176
N2*B*—H3*NB*···Cl3^ii^	0.92	2.32	3.231 (2)	171
O1*WA*—H1*WA*···Cl4^iv^	0.92	2.42	3.204 (2)	143
O1*WA*—H2*WA*···O1*A*^v^	0.91	1.89	2.799 (3)	173
O1*WB*—H1*WB*···O1*WA*^iv^	0.90	1.78	2.672 (3)	175
O1*WB*—H2*WB*···Cl2	0.91	2.33	3.231 (2)	169
C12*B*—H12*E*···O1*WA*^vi^	0.98	2.58	3.487 (3)	153
C3*B*—H3*BA*···O1*B*^vii^	0.95	2.56	3.373 (3)	143

Symmetry codes: (i) *x*, *y*−1, *z*; (ii) −*x*+1, *y*−1/2, −*z*+1/2; (iii) −*x*, *y*−1/2, −*z*+1/2; (iv) −*x*+1, −*y*+1, −*z*+1; (v) *x*+1, *y*, *z*; (vi) −*x*+1, *y*+1/2, −*z*+1/2; (vii) −*x*+1, −*y*+2, −*z*.
